# *Staphylococcus aureus* internalization impairs osteoblastic activity and early differentiation process

**DOI:** 10.1038/s41598-021-97246-y

**Published:** 2021-09-03

**Authors:** W. Mouton, J. Josse, C. Jacqueline, L. Abad, S. Trouillet-Assant, J. Caillon, D. Bouvard, M. Bouchet, F. Laurent, A. Diot

**Affiliations:** 1 CIRI - Centre International de Recherche en Infectiologie, Inserm, U1111, Université Claude Bernard Lyon 1, CNRS, UMR5308, École Normale Supérieure de Lyon, Université Lyon, Lyon, France; 2 Department of Bacteriology, Institute for Infectious Agents, Hospices Civils de Lyon, Lyon, France; 3 Regional Reference Centre for Complex Bone and Joint Infection (CRIOAc Lyon), Hospices Civils de Lyon, Lyon, France; 4 Faculté de Médecine, EA3826 « Thérapeutiques Cliniques et Expérimentales des Infections », Université de Nantes, Nantes, France; 5 CRBM, University of Montpellier, CNRS-5237, 1919 Route de Mende, 34293 Montpellier, France; 6 Institut De Génomique Fonctionnelle De Lyon, ENS de Lyon, UMR CNRS 5242, Lyon, France; 7 National Reference Centre for Staphylococci, Hospices Civils de Lyon, Lyon, France; 8 Department of Clinical Microbiology, Northern Hospital Group, Hospices Civils de Lyon, Lyon, France

**Keywords:** Bacteriology, Bacterial infection

## Abstract

*Staphylococcus aureus* is the most frequent aetiology of bone and joint infections (BJI) and can cause relapsing and chronic infections. One of the main factors involved in the chronicization of staphylococcal BJIs is the internalization of *S. aureus* into osteoblasts, the bone-forming cells. Previous studies have shown that *S. aureus* triggers an impairment of osteoblasts function that could contribute to bone loss. However, these studies focused mainly on the extracellular effects of *S. aureus*. Our study aimed at understanding the intracellular effects of *S. aureus* on the early osteoblast differentiation process. In our in vitro model of osteoblast lineage infection, we first observed that internalized *S. aureus* 8325-4 (a reference lab strain) significantly impacted *RUNX2* and *COL1A1* expression compared to its non-internalized counterpart 8325-4^∆*fnbAB*^ (with deletion of *fnbA* and *fnbB*). Then, in a murine model of osteomyelitis, we reported no significant effect for *S. aureus* 8325-4 and 8325-4^∆*fnbAB*^ on bone parameters at 7 days post-infection whereas *S. aureus* 8325-4 significantly decreased trabecular bone thickness at 14 days post-infection compared to 8325-4^∆*fnbAB*^. When challenged with two clinical isogenic strains isolated from initial and relapse phase of the same BJI, significant impairments of bone parameters were observed for both initial and relapse strain, without differences between the two strains. Finally, in our in vitro osteoblast infection model, both clinical strains impacted alkaline phosphatase activity whereas the expression of bone differentiation genes was significantly decreased only after infection with the relapse strain. Globally, we highlighted that *S. aureus* internalization into osteoblasts is responsible for an impairment of the early differentiation in vitro and that *S. aureus* impaired bone parameters in vivo in a strain-dependent manner.

## Introduction

*Staphylococcus aureus* is a successful opportunistic pathogen and commensal bacteria of humans colonizing 2 billion individuals worldwide^1^. It causes superficial or deep-seated suppurative infections including bone and joint infections (BJI). Due to its ability to colonize medical equipment, including catheters or prostheses, *S. aureus* is involved up to 75% of total BJI cases representing the most frequent aetiology^2,3^.

BJI can generate high inflammation and bone destruction. From a retrospective study performed in France in 2018 from data collected in 2013, mortality rate associated to BJI reached 5.2%, representing approximately 2,000 deaths/year in France^4^. With the ageing of the population and the increase of hip and knee prosthesis implantation (100,000 and 50,000 per year in France, respectively), elderly people are the most affected with infection rates ranging from 1 to 3% of total prosthesis implantation.

This is worrying as BJIs have frequent rate of relapses, estimated at 10–20% of cases on average and as high as 80% in severe situations, and therefore represent a major public health issue^5^. This chronicization of the infection has a major human and economic cost, up to 150 k€/episode due to longer hospital stays and antibiotics courses, with a risk of life-threatening complications for the patient. Chronic osteomyelitis is defined as a long-standing infection evolving over months or years and characterized by the persistence of microorganisms, low-grade inflammation and progressive bone tissue destruction^3^.

Three main factors were proposed to contribute to treatment failures: (i) the poor diffusion of antimicrobial agents within bone tissue^6^, (ii) biofilm formation on prosthetic materials^7^ and (iii) the internalization of bacteria into cells, notably osteoblasts, in which bacteria avoid extracellular antibiotics^8^ and escape the immune system^9^. Osteoblasts are bone cells involved in osteogenesis and regulation of bone resorption through their interaction with osteoclasts, the bone resorbing cells^10^. Their main function is to synthesize and mineralize bone matrix during skeletal growth and to renew or repair this matrix in adults. Osteoblasts differentiate from a pluripotent stem cells population to form proliferating preosteoblasts, these cells migrate and reach bony surfaces where they mature into bone depositing (mature) osteoblasts. Differentiation is divided into several stages (preosteoblast, immature osteoblast and mature osteoblast) characterized by the expression of specific transcription factors and functional markers^11^. Differentiation into pre-osteoblasts is characterized by the expression of the transcription factor RUNX2. Next, cells continue to proliferate and produce type I alpha 1 collagen (COL1A1) and osteopontin (OPN). Then, cells further differentiate and display a strong expression of bone matrix collagens such as *COL1A1* while the expression of *ALP* allows its mineralization. Finally, matrix mineralization occurs when the organic scaffold is enriched with osteocalcin (OCN) which promotes deposition of mineral substance^12^.

The internalization of *S. aureus* into osteoblasts and periprosthetic cells has been well-studied and intracellular staphylococci have been observed in clinical samples^13–15^. Although the process leading to internalization is not fully understood, it mainly involves an interaction between ß1integrin on the osteoblast side and the fibronectin-binding proteins (FnBPs) on the bacterial side, bridged together by fibronectin, an extracellular matrix protein^16–18^.

Previous studies have shown that *S. aureus* infecting osteoblasts can trigger (i) an increased production of cytokines responsible for a sustained and deleterious immune response (for a comprehensive review see^9^), (ii) an increased production of RANK-L which stimulates osteoclastogenesis^19^ (iii) an impairment of osteoblasts function (lower expression of alkaline phosphatase (ALP), type I collagen, osteocalcin) and mineralization capacity^19–21^ or (iv) cell death^19,21^. Each of these cellular responses can participate in bone loss alone or in combination.

However, these studies focused mainly on the extracellular effects using either strains unable to invade cells^19,22^, filtered supernatants^7^, fixed bacteria^22^, or an infection setting not specific for intracellular bacteria^20^.

Our study was aimed at understanding the specific intracellular effects of *S. aureus* on the osteoblast differentiation process. For this, we used the *S. aureus* 8325-4 and its isogenic mutant strains which is mutated on the *fnb* genes (8325-4^∆*fnbAB*^) and thereby incompetent for cellular internalization^23–25^. We also tested in our model a couple of two clinical strains isolated from initial and relapse phases of the same infection^26^. We assessed the effects of these infections both in vitro in a gentamicin/lysostaphin protection assay with immortalized murine pre-osteoblasts engaged in the differentiation process and in vivo in a murine osteomyelitis model^27^.

## Results

### Effect of *S. aureus* infection and internalization on osteoblastic differentiation in vitro

**Figure 1:**
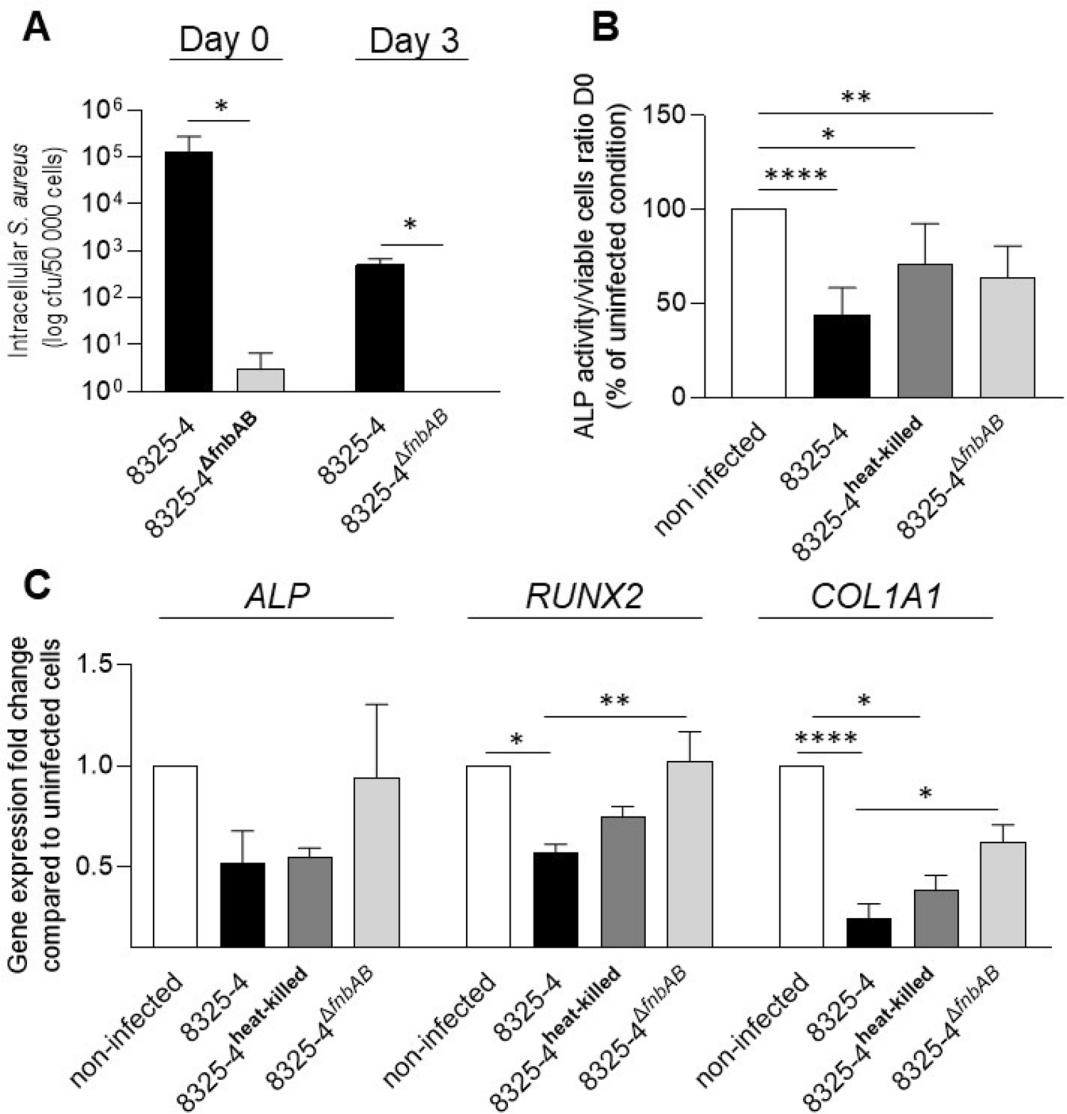
Effect of an infection by 8325–4 *S. aureus* strains on the early differentiation of OBß1 cells. (**A**) Quantification of internalization and intracellular survival of *S. aureus* 8325–4 and 8325–4^∆*fnbAB*^ into OBß1 osteoblasts. (**B**) Effect of *S. aureus* 8325–4, 8325-4^heat killed^ and 8325–4^∆*fnbAB*^ on ALP activity. Results are expressed as a proportion of the activity detected for the non-infected condition. (**C**) Effect of *S. aureus* 8325–4, 8325-4^heat killed^ and 8325–4^∆*fnbAB*^ on the expressions of *ALP*, *RUNX2*, *COL1A1*, expressed as a fold change compared to the non-infected condition. At least 2 independent experiments in technical duplicates were performed. *, ** or **** mean *P* < 0.05, *P* < 0.01 and *P* < 0.0001 respectively.

First, osteoblast cells OBß1 were infected with *S. aureus* 8325-4 or its internalization incompetent isogenic mutant (*S. aureus* 8325-4^∆*fnbAB*^) to validate our model. After 3 h (Day 0), *S. aureus* 8325-4 strain was internalized with a mean of 1,29 × 10^5^ CFU whereas the 8325-4^∆*fnbAB*^ was internalized with a mean of 2,91 CFU (Fig. 1A). At Day 3, only 8325-4 survived intracellularly (Fig. 1A).

Then, we investigated the effect of 8325-4, 8325-4^∆*fnbAB*^ or 8325-4^heat killed^ on the ALP activity in osteoblasts. Dead *S. aureus* can be internalized by osteoblasts, but they provoke a lower host response when compared to their live counterpart *S. aureus*^28,29^. The three strains significantly impaired the ALP activity of osteoblasts, without significant difference between the three infected conditions but with a higher p value for the internalized live strain *S. aureus* 8325-4 (Fig. 1B). To further analyse how internalization of *S. aureus* could impact osteoblast differentiation, we analysed the expression of *ALP, RUNX2* and *COL1A1* by RTqPCR. *ALP* expression was not significantly different from the non-infected cells, whatever the observed infected condition. However, *RUNX2* expression was significantly impaired by 8325-4 but not by 8325-4^∆*fnbAB*^ nor 8325-4^heat-killed^ (Fig. 1C). Impact of *S. aureus* 8325-4 on *RUNX2* expression was significantly different compared to its non-internalized counterpart 8325-4^∆fnbAB^ (Fig. 1C). Expression of *COL1A1* was significantly decreased following infection with 8325-4 and 8325-4^heat-killed^. Effect on *COL1A1* expression was significantly different between 8325-4 and 8325-4^∆*fnbAB*^ (Fig. 1C).

Overall, lack of internalization and intracellular survival appeared to abrogate the effect of *S. aureus* 8325-4 on *RUNX2* and *COL1A1* but did not significantly impact the expression or activity of ALP.

### Effect of *S. aureus* infection and internalization on bone in vivo

We adapted the protocol from Cassat et al*.*^27^ to infect the femur of mice for 7 and 14 days with 8325-4 and 8325-4^∆*fnbAB*^. After microtomography and 3D reconstruction of the bone (Suppl. Fig. S1), we quantified the mineral content (BMC) and density (BMD) of the trabecular bone as well as the number, spacing and thickness of the trabeculae. The cortical bone was excluded from analysis due to the cortical bone defect created for the infection.

**Figure 2:**
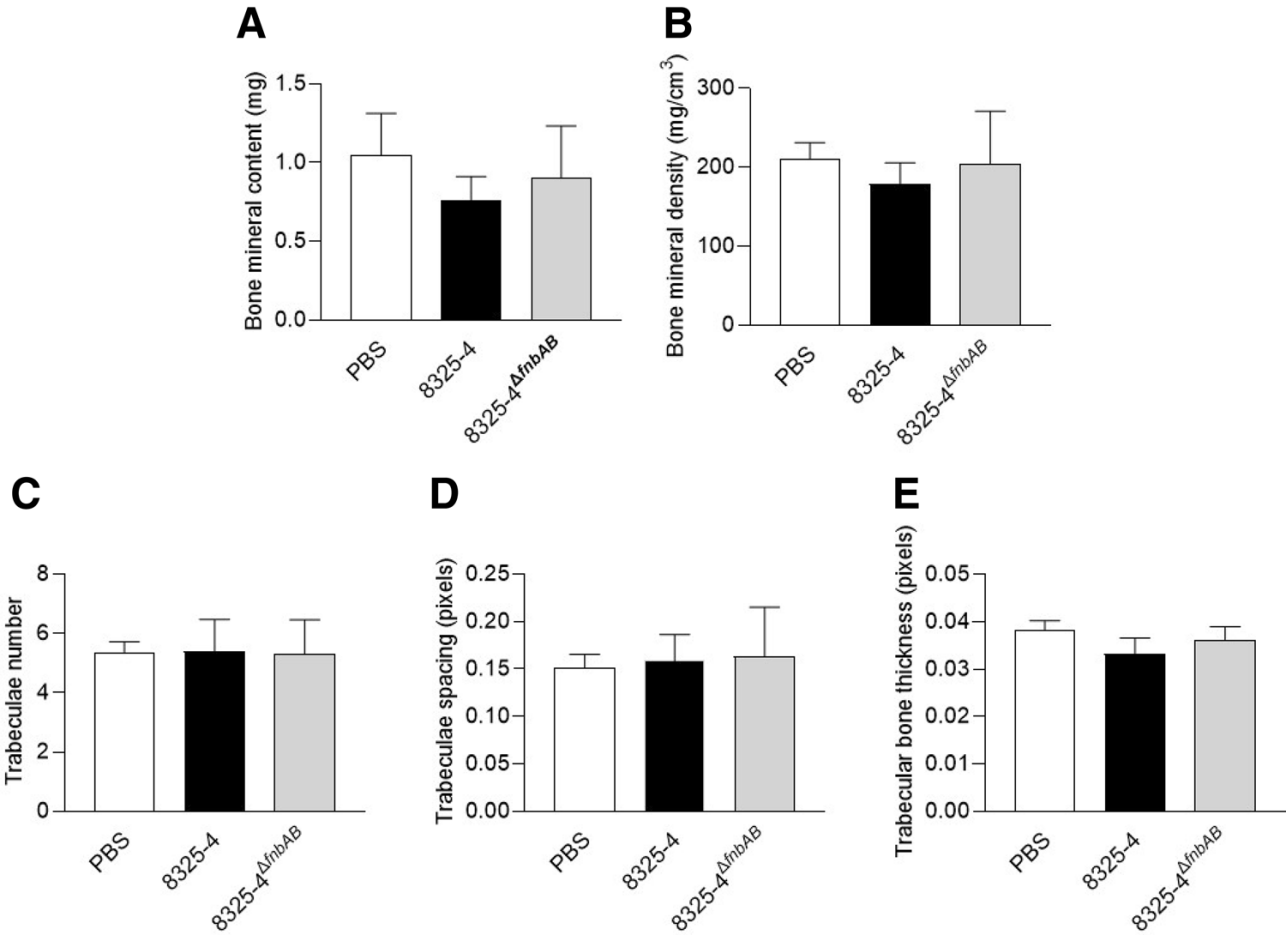
Effect of *S. aureus* 8325–4 strain on the trabecular bone in vivo 7 days after infection. The mice were infected at the midfemur by trepanation with a 20-gauge needle. Controls were realized using a PBS injection (PBS, n = 4) and infection were carried out using the invasive-competent *S. aureus* 8325–4 strain (n = 13) or invasive-incompetent *S. aureus* 8325–4^∆f*nbAB*^ strain (n = 13). After microtomography and 3D reconstruction, the following parameters were quantified: bone mineral content in mg (**A**), bone mineral density in mg/cm3 (**B**), trabeculae number in the ROI (**C**), space between the trabeculae in pixels (**D**) and trabecular bone thickness in pixels (**E**).

After 7 days of infection, we did not observe any difference between PBS (control without infection), infection with 8325-4 or infection with 8325-4^∆f*nbAB*^ (Fig. 2).

**Figure 3:**
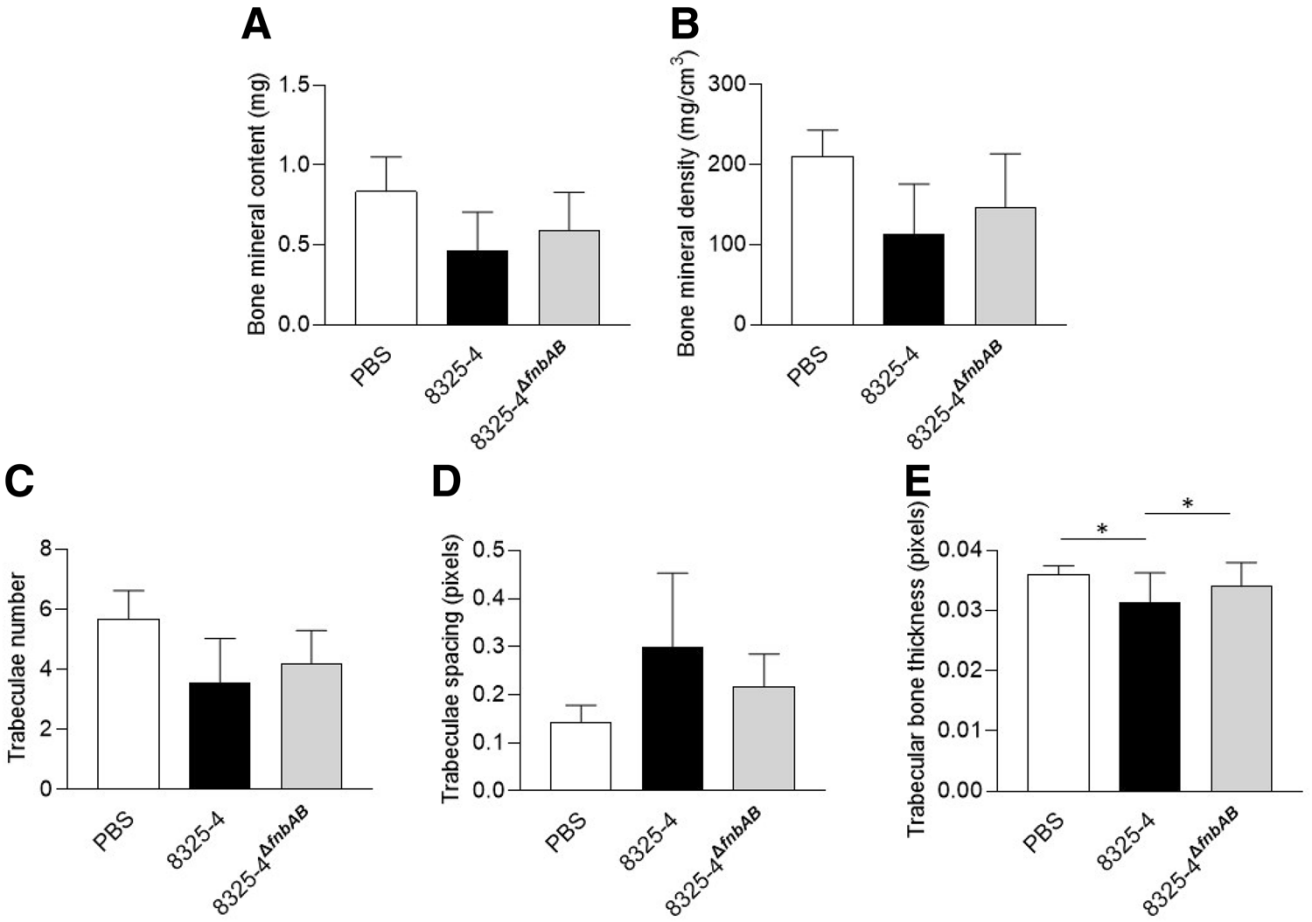
Effect of *S. aureus* 8325–4 strain on the trabecular bone in vivo 14 days after infection. A prolonged infection was performed for 14 days with the same conditions as in Fig. 6 (PBS, n = 3; 8325–4, n = 6; 8325–4^∆f*nbAB*^, n = 6). After microtomography and 3D reconstruction, the following parameters were quantified: bone mineral content in mg (**A**), bone mineral density in mg/cm3 (**B**), trabeculae number in the ROI (**C**), space between the trabeculae in pixels (**D**) and trabecular bone thickness in pixels (**E**). * means *P* < 0.05.

When the infection was prolonged for 14 days, we did not observe significant variation for BMC, BMD, trabecular number or trabecular spacing. However, trabecular thickness was significantly impact by *S. aureus* 8325-4 when compared to PBS control, but not by 8325-4^∆fnbAB^ (Fig. 3E). Moreover, the effect of 8325-4 on bone thickness was significantly different than observed with 8325-4^∆f*nbAB*^ (Fig. 3E).

### Effect of clinical *S. aureus* strains on bone in vivo

Previous in vitro data suggest that internalization has a major role in the inhibition of osteogenesis. Therefore, we tried to correlate virulence with bone loss using more invasive strains. We challenged our in vivo model with strains isolated during BJI and chose to investigate the effects of 2 isogenic strains isolated during the initial and relapse phase from the same infection^26^. After microtomography and 3D reconstruction of the bone (Suppl. Fig. S2), we quantified the same parameters that we used previously.

**Figure 4:**
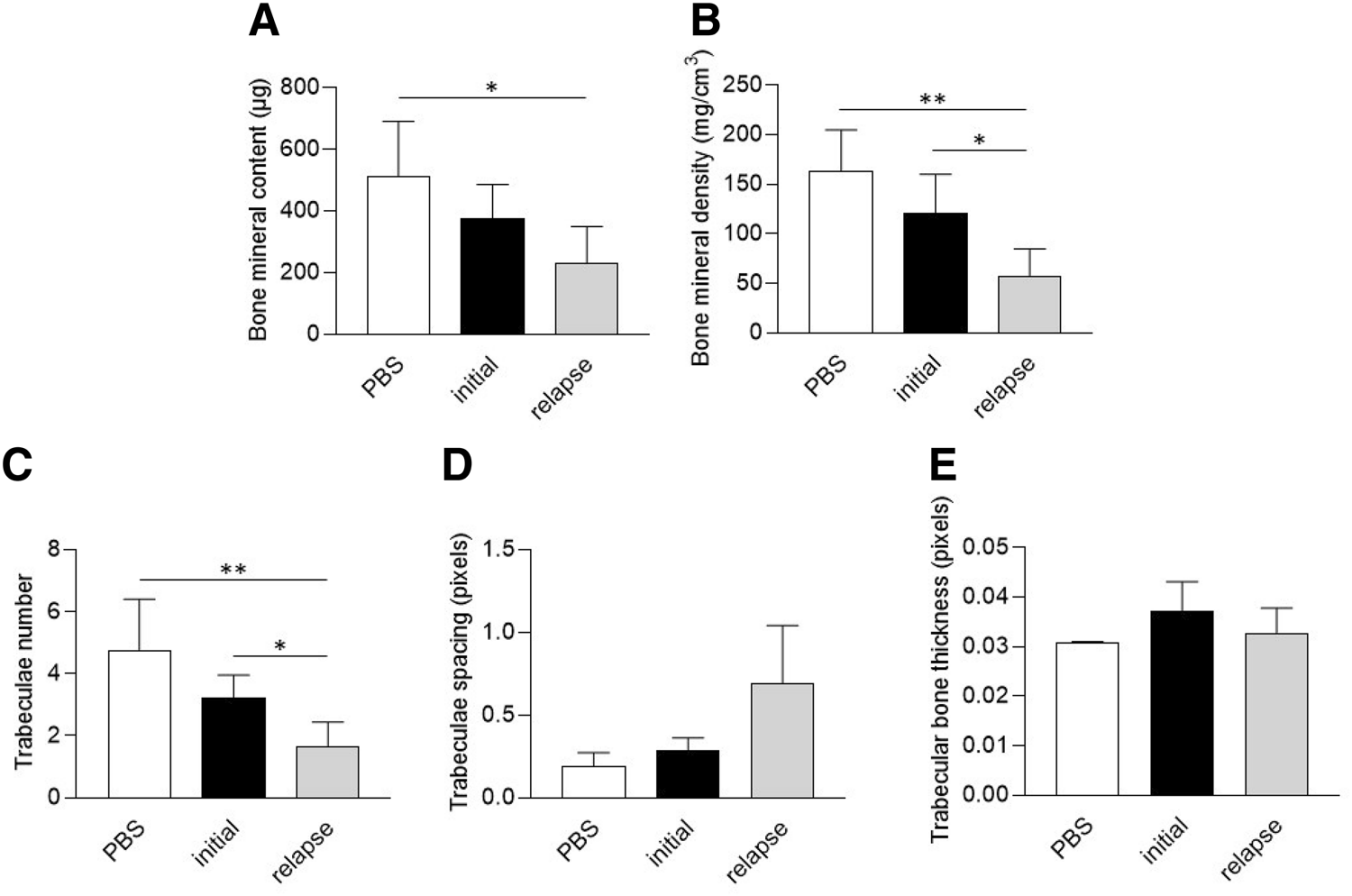
Effect of *S. aureus* clinical strains (initial and relapse) on the trabecular bone in vivo after 7 days of infection. Mice were infected at the midfemur using a clinical strain isolated during the initial (Initial, n = 5) or relapse phase of a bone infection (Relapse, n = 5) or PBS injection as control (PBS, n = 3). After microtomography and 3D reconstruction, the following parameters were quantified: bone mineral content in µg (**A**), bone mineral density in mg/cm3 (**B**), trabeculae number in the ROI (**C**), space between the trabeculae in pixels (**D**) and trabecular bone thickness in pixels (**E**). * and ** mean *P* < 0.05 and *P* < 0.01 respectively.

After 7 days, BMC was significantly reduced after infection with the relapse strain when compared to the PBS condition (Fig. 4A). For BMC and trabecular number, both initial and relapse strain had an effect, with a higher p value for the relapse strain (Fig. 4B,C). Trabecular spacing and trabecular thickness were not significantly impacted by neither initial nor relapse strains (Fig. 4D,E).

**Figure 5:**
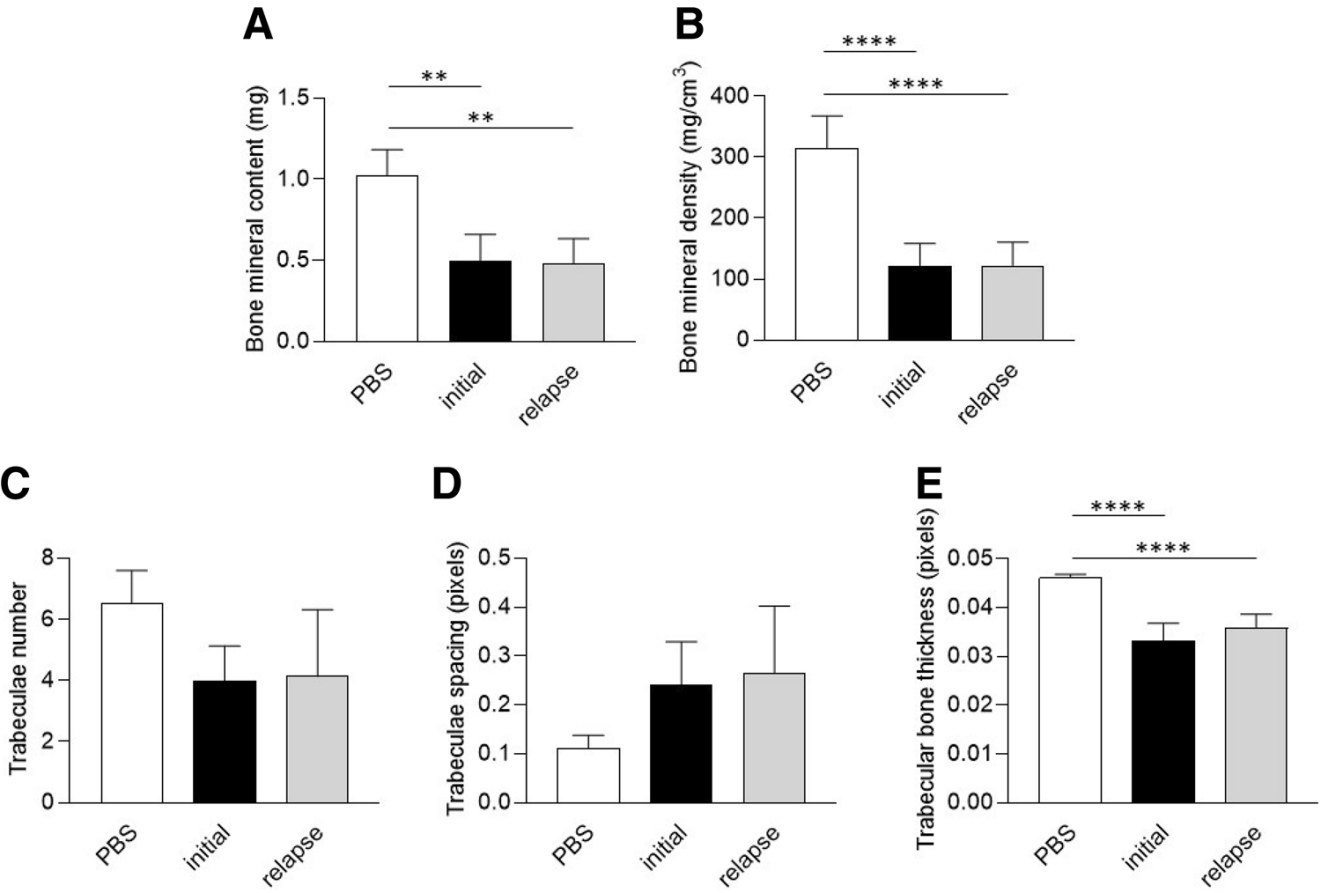
Effect of *S. aureus* clinical strains (initial and relapse) on the trabecular bone in vivo after 14 days of infection. A prolonged infection was performed for 14 days with the same conditions as in Fig. 1 (PBS, n = 3; Initial, n = 7; Relapse, n = 6). After microtomography and 3D reconstruction, the following parameters were quantified: bone mineral content in mg (**A**), bone mineral density in mg/cm3 (**B**), trabeculae number in the ROI (**C**), space between the trabeculae in pixels (**D**) and trabecular bone thickness in pixels (**E**). ** and **** mean *P* < 0.01 and *P* < 0.0001 respectively.

After 14 days, both initial and relapse strains significantly decreased the BMC and BMD, without difference (Fig. 5A,B). Trabecular number and trabecular spacing were not impacted by the infection (Fig. 5C,D). However, trabecular thickness was significantly decreased after infection when compared to PBS control, with both initial and relapse strains (Fig. 5E).

Globally, no significant difference was observed between the initial and the relapse strain in our murine model.

### Effect of clinical *S. aureus* strains on osteoblastic differentiation in vitro

**Figure 6:**
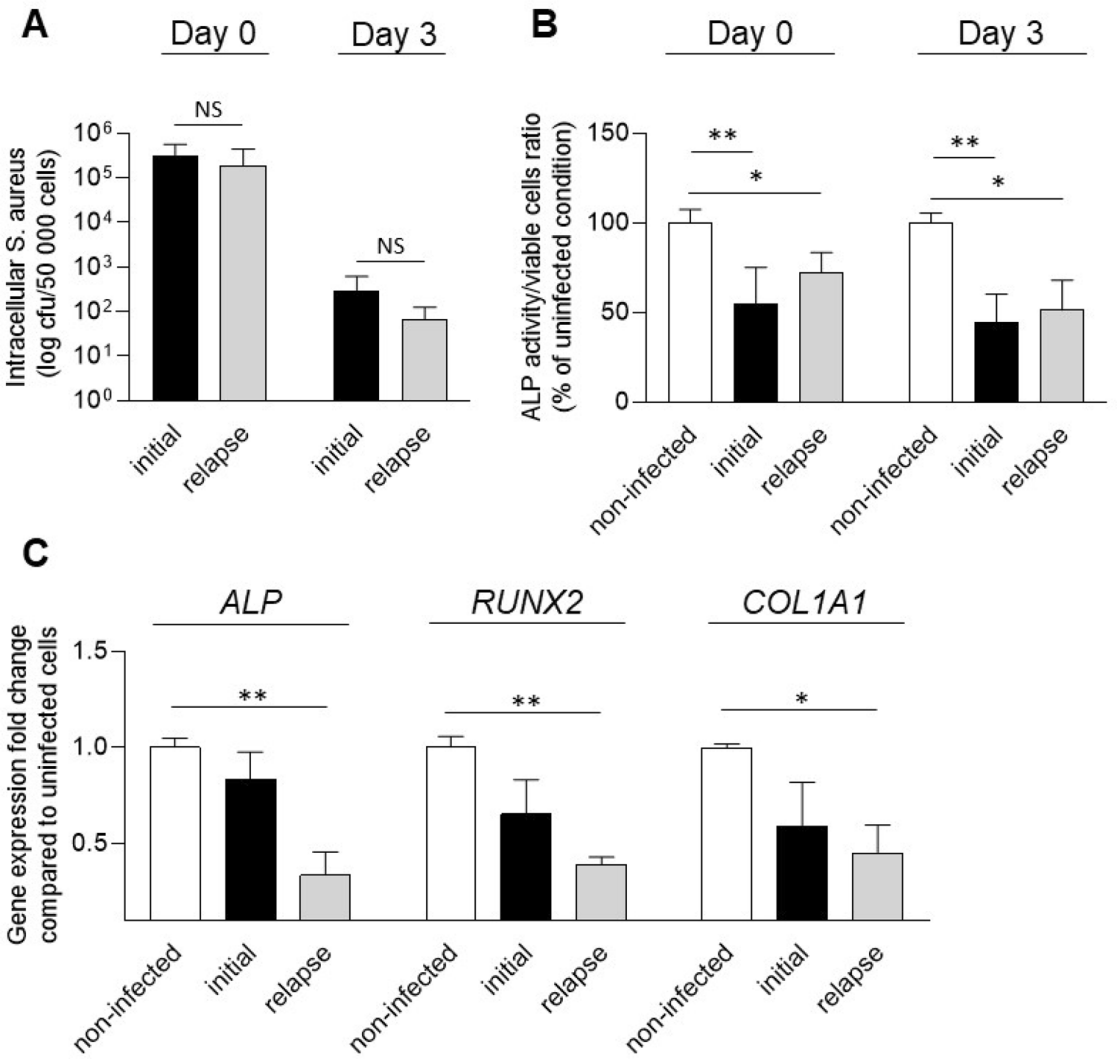
Effect of *S. aureus* clinical strains (initial and relapse) on the early differentiation of OBß1 cells. (**A**) Quantification of internalization and intracellular survival of *S. aureus* 8325–4 and 8325–4^∆fnbAB^ into OBß1 osteoblasts. (**B**) Effect of *S. aureus* 8325–4, 8325-4^heat killed^ and 8325–4^∆fnbAB^ on ALP activity. Results are expressed as a proportion of the activity detected for the non-infected condition. (**C**) Effect of *S. aureus* 8325–4, 8325-4^heat killed^ and 8325–4^∆fnbAB^ on the expressions of *ALP*, *RUNX2*, *COL1A1*, expressed as a fold change compared to the non-infected condition. At least 2 independent experiments in technical duplicates were performed. * and ** mean *P* < 0.05 and *P* < 0.01 respectively.

In our in vitro setup, both initial and relapse strains were able to invade osteoblasts and survive intracellularly, without significant difference between strains (Fig. 6A). Both strains significantly impaired ALP activity 3 h post-infection (Day 0) and 3 days post-infection (Fig. 6B). Concerning the expression of *ALP*, *RUNX2* and *COL1A1*, significant decreases were observed only for the relapse strain when compared to the non-infected control, without significant difference between the initial and the relapse strains (Fig. 6C).

## Discussion

Bone tissue homeostasis relies on the balance between osteoblast mineralization and osteoclast resorption activities. Acute BJIs due to *S. aureus* are characterized by bone loss mostly due to the cytotoxic activity of bacteria^27,30^ combined with an increased activity of osteoclasts^22,31^. However, chronic staphylococcal BJIs are less aggressive and slowly progress. They lead to bone demineralization and the loosening of the prosthetic material responsible for an unsettled long-term outcome for patients. The chronicization of infections relies on *S. aureus* ability to shelter from the immune system as well as antibiotics, mainly through biofilm formation or intracellular survival^7,9^.

In this work, our in vitro results suggested that *S. aureus* internalization partially impaired the early differentiation of osteoblasts. ALP expression and activity were not significantly impacted by the absence of internalization and intracellular survival of *S. aureus* 8325-4^∆*fnbAB*^ when compared to the wildtype strain 8325-4 (Fig. 1B,C). However, significant differences were observed for *RUNX2* and *COL1A1* expression. Only the internalized 8325-4 impacted the expressions of *RUNX2* and *COL1A1* whereas no impact was observed for the non-internalized 8325-4^∆*fnbAB*^ (Fig. 1C). It suggests that internalization and intracellular survival impact the expression of these two genes. Impact of *S. aureus* on *RUNX2* expression was already observed after stimulation of osteoblasts with biofilm-conditioned medium or in organ cultured mouse parietal bones^7,32^. However, the impact of *S. aureus* internalization and intracellular survival on the expression of osteoblastic genes has not been widely investigated. Our hypothesis is that internalization of *S. aureus* through the FnBPs / fibronectin / integrin α5β1 could impact the osteoblast activity through modulation of the β1 integrin activity. Indeed, stimulation of β1 integrin can modulate the osteoblast activity and *RUNX2* transcription^33,34^. Through internalization, *S. aureus* could interfere with the integrin β1 / RUNX2 pathway and decrease the expression of *RUNX2* and *COL1A1* as type I collagen is regulated by RUNX2^35^.

Concerning our in vivo experiments with *S. aureus* 8325-4 and 8325-4^∆*fnbAB*^, we observed significant differences only for trabecular bone thickness at 14 days post-infection (Fig. 3E). Lower bone thickness after infection with the wildtype *S. aureus* 8325-4 but not with the non-internalized 8325-4^∆*fnbAB*^ appears coherent with our in vitro results. Indeed, if 8325-4 impacts the expression of *RUNX2* and *COL1A1*, it could at least impact the bone formation that normally counterbalances the bone resorption by osteoclasts, finally leading to a decrease of bone thickness. *S. aureus* 8325-4 also seemed to decrease BMC, BMD, trabecular number and increase trabecular spacing more than its non-internalizing counterpart 8325-4^∆*fnbAB*^ at 14 days post-infection (Fig. 3). However, no significant difference was observed, potentially due to the too small number of animals used in this study.

Similar effects were observed when we tested the clinical strains in our in vitro model (Fig. 6). ALP activity was affected in a similar way after infection with the initial or the relapse strain 6B). However, only the relapse strain significantly decreased the *ALP*, *RUNX2* and *COL1A1* gene expressions (Fig. 6C). However, these differences cannot be explained a difference of internalization or intracellular survival between initial and relapse strains as no difference was detected the in vitro setting used in this study (Fig. 6A). This point was surprising for us. Indeed, we initially chose this couple of initial/relapse strains because the relapse strain shown a better ability to survive intracellularly in one of our previous study. In this previous study, Trouillet-Assant et al.observed that the relapse strain is less cytotoxic and survive better intracellularly than its initial counterpart^26^. In the current study, we did not detect any differences in terms of cytotoxicity (data not shown) or intracellular survival between the 2 clinical strains. However, Trouillet-Assant et al*.* used a different type of cells (human primary osteoblasts) and a higher MOI. Indeed, they used a MOI of 250:1 which induces high cell death whereas in the current study we chose a MOI of 50:1 to avoid that cytotoxicity parasitizes our observations regarding osteoblastic activities. Higher ability of the relapse strain to survive intracellularly could be directly related to its lower cytotoxicity compared to the initial strain, preserving its intracellular reservoir. In experiments with lower MOI, absence of cell death could smooth the differences of intracellular survival between the initial and the relapse strains, resulting in no difference regarding intracellular survival.

In the in vivo model, both the initial and relapse strains impacted various parameters with specificities regarding the time of infection. However, no significant difference wase observed between the two strains. This could be explained by the fact that both strains were able to be internalized by osteoblasts and impact the osteoblastic activities.

Overall, we highlighted in this study that *S. aureus* internalization into osteoblasts is responsible for an impairment of the early differentiation in vitro and that *S. aureus* impaired bone parameters in vivo, in a strain-dependent and time-dependent manner. However, it is difficult to affirm that the internalization is directly involved in the in vivo observed phenomena. Investigating in an in vivo environment how internalization and intracellular survival of *S. aureus* inside osteoblasts impact osteoblast activity seems necessary to fully understand the pathogenic mechanisms of BJI.

## Materials and methods

### Bacterial strains

Isogenic strains of *S. aureus* 8325-4 and 8325-4^∆*fnbAB*^ were used in this study and are routinely used in our lab^23–25^. The latter was genetically deleted for the *fnbA* and *fnbB* genes and its internalization in osteoblasts is strongly inhibited^23^. Construction was performed by Greene et al.^36^. We also used a couple of clinical *S. aureus* strains isolated during the initial and relapse phases of an PJI in the same patient (patient 3 described in^26^).

### Cell lines and culture conditions

The pre-osteoblastic OBβ_1_^+/+^ cell line, obtained after immortalization of mouse primary cells with the T viral oncogene of SV40, were cultured in 75cm^2^ flasks (T75, BD Falcon™, Franklin Lakes, NJ, USA) at 37 °C under 5% CO_2_, in a complete culture medium composed of Dulbecco's Modified Eagle Medium (DMEM) and supplemented with L-glutamine, 10% fetal calf serum (FCS), penicillin (100 μg/mL), and streptomycin (100 μg/ml) (all from Gibco, Carlsbad, CA, USA). Functional osteoblasts were obtained in vitro after plating in a 24-well plate at a density of 50,000 cells per well and culture for 3 days to obtain a close to 100% of confluence uniform cell layer. Then, the differentiation was triggered using αMEM (Gibco,Carlsbad, CA, USA) supplemented with L-Glutamine (2 mM), ascorbic acid (50 µg/mL), and β-Glycerophosphate (10 mM), and renewed every 3 days (all from Sigma-Aldrich, Saint-Louis, MO, USA).

### Infection and Gentamicin / Lysostaphin protection assay

On the day of the differentiation induction, the cells were infected with the indicated strains at a MOI (multiplicity of infection, i.e. the number of bacteria per cell) of 50:1. After 2 h of contact, adherent and non-adherent extracellular bacteria were eliminated with 200 µg/mL of gentamicin (Gibco, Carlsbad, CA, USA) for 1 h. Fresh media supplemented with 40 µg/mL gentamicin and 10 µg/mL lysostaphin (Sigma-Aldrich, Saint-Louis, MO, USA) was then added for further culture. At 3 h, 72 h and 120 h post-infection, the intracellular bacteria load was assessed by osmotic cell lysis of 2 wells and plating (Easy Spiral Interscience, Saint-Nom-la-Breteche, France) of the lysates on TSA agar plates. CFU were counted after 18-24 h at 37 °C. CFU results were normalized for 50 000 cells. This experimental set up was also used for the alkaline phosphatase experiments, the cell viability tests and RT-qPCR experiments.

### Alkaline phosphatase measurement

The cells were washed twice with PBS and lysed with 1 mL of PBS + 0.1% Triton-X100, at the indicated times. The lysate was sonicated for 3 min, vortexed and centrifuged at 14,000 rpm for 3 min. ALP activity was quantified from recovered supernatants on a Vista 3000 (Siemens Health care, Erlangen, Germany). Briefly, The ALP method employs alkaline phosphatase that catalyzes the transphosphorylation of pnitrophenyiphosphate (p-NPP) to p-nitrophenol (p-NP). The change in absorbance at 405nmm due to the formation of p-NP is directly proportional to the ALP activity^37,38^.

### Cell viability by the MTT method

The cell viability was evaluated using MTT (Methylthiazolyldiphenyl-tetrazolium bromide) (Sigma-Aldrich, Saint-Louis, MO, USA) measurement, following the manufacturer’s recommendations. Briefly, the cells were washed twice with 700μL of αMEM before addition of 400μL of working MTT solution to each well. After 1 h of incubation at 37 °C, the solution was removed and 200μL/well of isopropanol acid was used to solubilize the formazan. The OD_450nm_ was measured in a round-bottom 96-well plate, with a TECAN Infinite® Pro 200 (TECAN, Austria).

### RNA extraction

Total RNA from the cells were extracted using the RNeasy minikit (Qiagen, Hilden, Germany) according to the manufacturer’s recommendations, with a DNAse I treatment applied on the column before the last washing steps. Briefly, Between the first and the second wash step ; we added the equivalent 1 µl of DNAse I / per 1µL of RNA following manufacturer recommendation (Invitrogen™ DNase I, Amplification Grade) directly on the column membrane and incubate for 10 min at room temperature. Quality of RNA extraction was measured on a Nanodrop 8000 (Thermo Scientific, Waltham, MA, USA).

### RT-qPCR analysis

Reverse transcription from total RNA was performed with a Reverse Transcription System kit (Promega, Madison, WI, USA) according to the manufacturer’s recommendations.

The qPCR was done using the LightCycler® FastStart DNA SYBR Green Kit in a LightCycler® Real-Time PCR System (both from Roche Diagnostic, Meylan, France). The primers used are described in the Table 1. The expression of each gene was normalized to the reference gene RanBP1 and calculated using the 2^−Δ∆Ct^ method^33^.

**Table 1:** Primers used for RTqPCR analysis.

Name	Forward 5ʹ–3ʹ	Reverse 5ʹ–3ʹ
ALP	GCCCTCTCCAAGACATATA	CCATGATCACGTCGATATCC
RUNX2	CCGCACGACAACCGCACCAT	CGCTCCGGCCCACAAATCTC
COL1A	CCTGGTAAAGATGGGCC	CACCAGGTTCACCTTTCGCACC
RanBP1	CGAGGACCATGATACTTCCACA	CCTCCAGCGTTTTAATTTCTTGC

### Murine Model of Osteomyelitis

The infection protocol is adapted from Cassat et al.^27^. The bacterial inoculum was prepared as described, washed twice with PBS, and resuspended to a concentration of 1.10^9^ CFU/mL in PBS. Animals were maintained in specific-pathogen-free conditions (group-housed) at the UTE-IRS2 Nantes Biotech Animal Facility (Nantes, France) following institutional guidelines.

Experimental procedure was approved by the Animal Ethics Committee of the Pays de la Loire and the French Ministry Higher Education, Research, and Innovation (APAFIS no. 2015072711421399). Female mice (RjOrl:SWISS, 6-week-old) received 0.1 mg/kg buprenorphine via subcutaneous injection and anesthesia was accomplished with a ketamine/xylazine mixture (IP administration 80 and 10 mg/kg respectively). The right hindlimb was shaved, disinfected and the mid-femur was exposed to create, by trepanation with a 20-gauge needle, a 1 mm diameter unicortical bone defect. Bacterial inoculum (2µL, approximately 2 × 106 CFU) was delivered using a Hamilton glass syringe through the bone defect into the intramedullary canal. Muscle fasciae and skin were sutured, and mice allowed to recover from anesthesia. Buprenorphine was administered every 12 h for 72 h postoperatively and as needed thereafter. Infection could proceed for 7 and 14 days, at which times mice were euthanized and the right femur was removed for microCT analysis. This study was carried out following animal care standard, in compliance with the ARRIVE guidelines^39^.

### X-ray microtomography bone analyses

The bone microCT pictures were acquired in pairs in a 1.5 mL tube containing 70% ethanol and a piece of hydroxyapatite for quantitation, using a Phoenix nanotom S scan (GE, Boston, MA, USA). The scan was set to a voltage of 100 kV and a current of 70μA, 3000 images were taken in 2 h and the size of the voxel was 5 μm. The bones were reconstructed using the Phoenix datosx rec software. We used the Microview software to measure the bone mineral density, bone mineral content, trabecular number, trabecular space and trabecular thickness of the trabecular bone.

### Graphical representation and statistical analysis

All graphics for in vitro experiments result from at least 2 independent experiments performed in technical duplicate (4 values). For in vivo experiments, number of animals per group is specified in figure captions. Results are presented as histograms “mean + SD”. The number of independent experiments or the number of animals used for each method is indicated in the caption for each figure. Due to the number of values, we chose to perform non-parametric statistical analysis. Mann–Whitney tests were performed for internalization and intracellular survival. Kruskal–Wallis tests followed by Dunn’s multiple comparisons tests were performed for the other experiments with more than two groups to compare. All analyses were performed using Prism software (GraphPad, San Diego, CA, USA).

## Supplementary Information

Supplementary Information.
