# Supplementary material for: Staphylococcus aureus internalization impairs osteoblastic activity and early differentiation process
Source: Sci Rep. 2021 Sep 3;11:17685. doi: 10.1038/s41598-021-97246-y (PMC8417294; doi:10.1038/s41598-021-97246-y)
Supplement: Supplementary file 1 — Supplementary Information. [file 41598_2021_97246_MOESM1_ESM.pptx]

## Slide 1
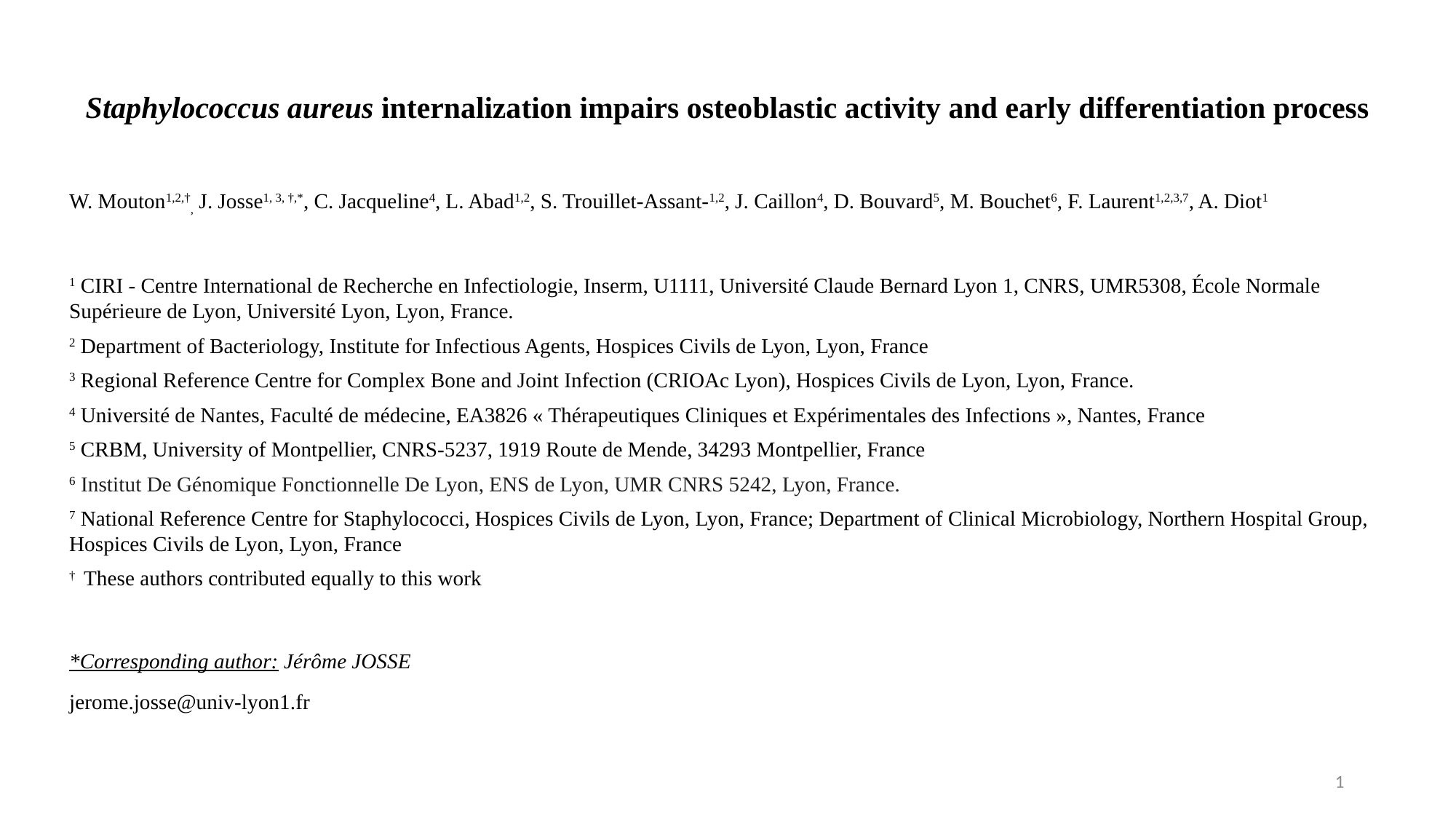

Staphylococcus aureus internalization impairs osteoblastic activity and early differentiation process
W. Mouton1,2,†, J. Josse1, 3, †,*, C. Jacqueline4, L. Abad1,2, S. Trouillet-Assant-1,2, J. Caillon4, D. Bouvard5, M. Bouchet6, F. Laurent1,2,3,7, A. Diot1
1 CIRI - Centre International de Recherche en Infectiologie, Inserm, U1111, Université Claude Bernard Lyon 1, CNRS, UMR5308, École Normale Supérieure de Lyon, Université Lyon, Lyon, France.
2 Department of Bacteriology, Institute for Infectious Agents, Hospices Civils de Lyon, Lyon, France
3 Regional Reference Centre for Complex Bone and Joint Infection (CRIOAc Lyon), Hospices Civils de Lyon, Lyon, France.
4 Université de Nantes, Faculté de médecine, EA3826 « Thérapeutiques Cliniques et Expérimentales des Infections », Nantes, France
5 CRBM, University of Montpellier, CNRS-5237, 1919 Route de Mende, 34293 Montpellier, France
6 Institut De Génomique Fonctionnelle De Lyon, ENS de Lyon, UMR CNRS 5242, Lyon, France.
7 National Reference Centre for Staphylococci, Hospices Civils de Lyon, Lyon, France; Department of Clinical Microbiology, Northern Hospital Group, Hospices Civils de Lyon, Lyon, France
† These authors contributed equally to this work
*Corresponding author: Jérôme JOSSE
jerome.josse@univ-lyon1.fr
1

## Slide 2
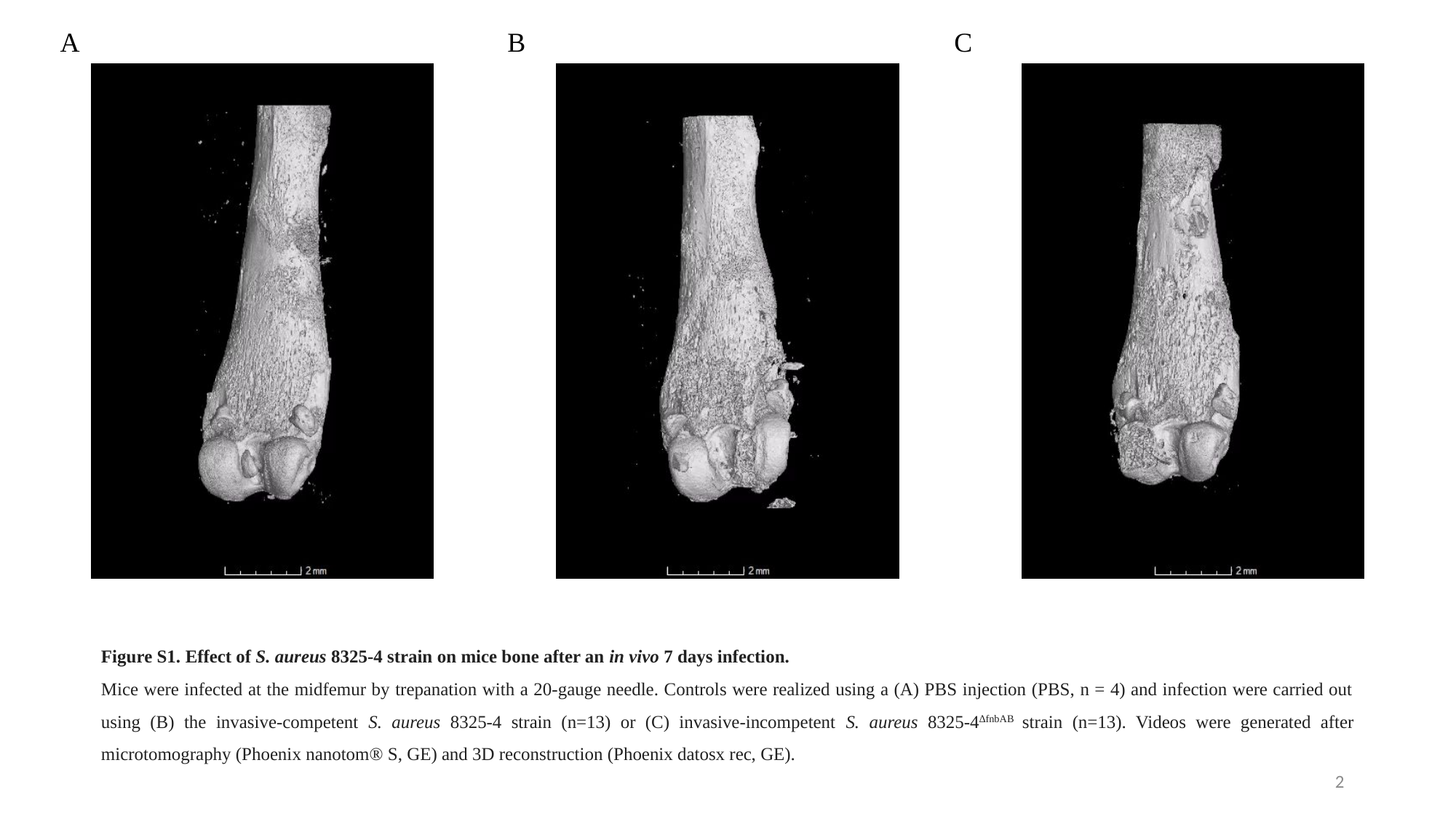

A
B
C
Figure S1. Effect of S. aureus 8325-4 strain on mice bone after an in vivo 7 days infection.
Mice were infected at the midfemur by trepanation with a 20-gauge needle. Controls were realized using a (A) PBS injection (PBS, n = 4) and infection were carried out using (B) the invasive-competent S. aureus 8325-4 strain (n=13) or (C) invasive-incompetent S. aureus 8325-4∆fnbAB strain (n=13). Videos were generated after microtomography (Phoenix nanotom® S, GE) and 3D reconstruction (Phoenix datosx rec, GE).
2

## Slide 3
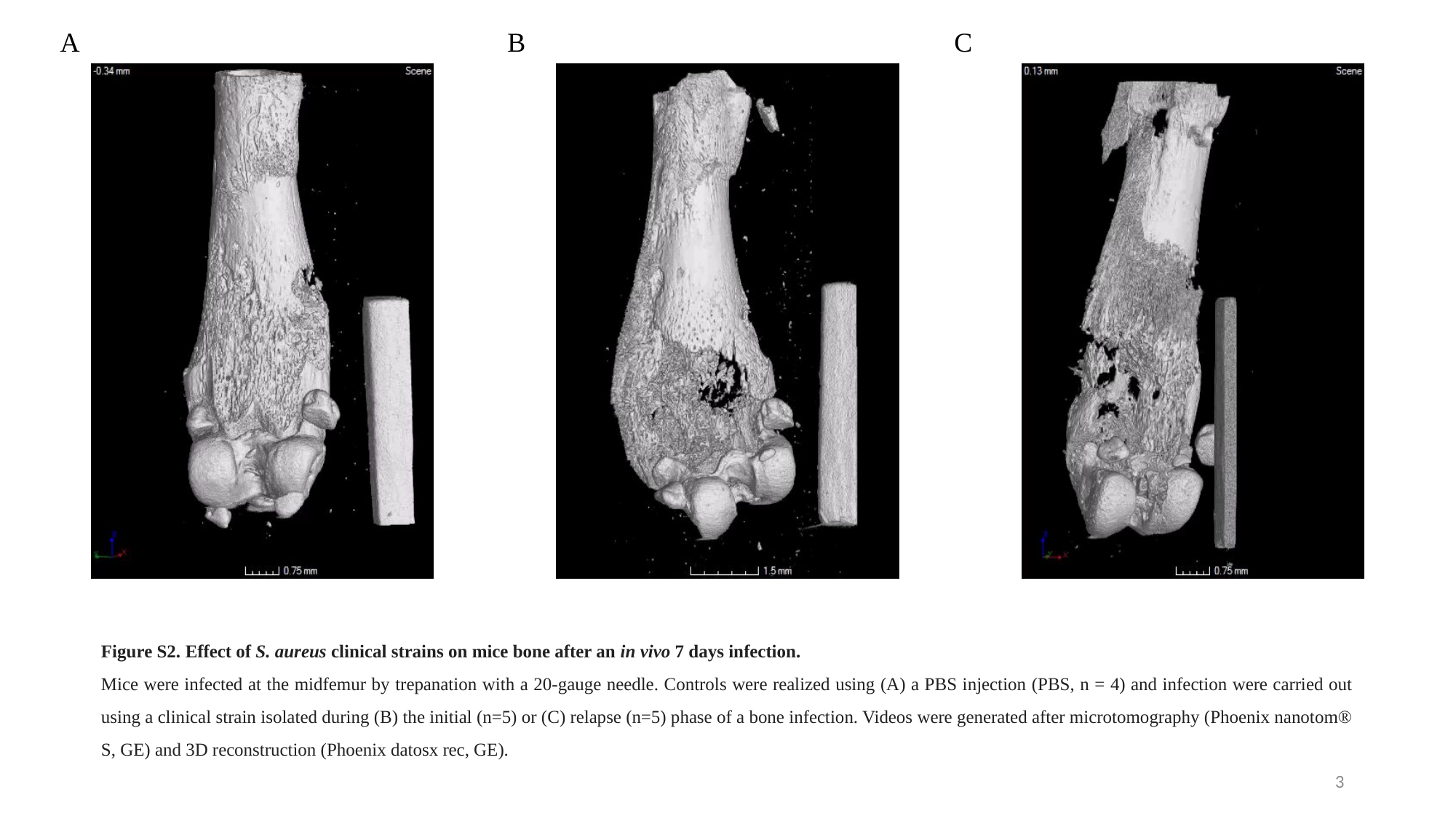

A
B
C
Figure S2. Effect of S. aureus clinical strains on mice bone after an in vivo 7 days infection.
Mice were infected at the midfemur by trepanation with a 20-gauge needle. Controls were realized using (A) a PBS injection (PBS, n = 4) and infection were carried out using a clinical strain isolated during (B) the initial (n=5) or (C) relapse (n=5) phase of a bone infection. Videos were generated after microtomography (Phoenix nanotom® S, GE) and 3D reconstruction (Phoenix datosx rec, GE).
3
